# Compact silicon multimode waveguide spectrometer with enhanced bandwidth

**DOI:** 10.1038/srep43454

**Published:** 2017-03-14

**Authors:** Molly Piels, Darko Zibar

**Affiliations:** 1 Technical University of Denmark, Department of Photonics Engineering, Kgs. Lyngby, 2800, Denmark

## Abstract

Compact, broadband, and high-resolution spectrometers are appealing for sensing applications, but difficult to fabricate. Here we show using calibration data a spectrometer based on a multimode waveguide with 2 GHz resolution, 250 GHz bandwidth, and a 1.6 mm × 2.1 mm footprint. Typically, such spectrometers have a bandwidth limited by the number of modes supported by the waveguide. In this case, an on-chip mode-exciting element is used to repeatably excite distinct collections of waveguide modes. This increases the number of independent spectral channels from the number of modes to this number squared, resulting in an extension of the usable range.

Spectrometers have wide applications in sensing and monitoring. Integrated spectrometers are especially interesting because they can be manufactured with low cost and footprint, which increases the range of potential applications. However, it is very difficult to manufacture a high-resolution spectrometer with a high dynamic range using a traditional echelle or arrayed waveguide grating. This is because the resolution is inversely proportional to the footprint, and increasing the footprint results in increased variation in physical dimensions, which in turn causes phase errors that reduce the dynamic range. As a result, most integrated high resolution (<50 GHz) spectrometers demonstrated to date have had dynamic ranges (crosstalk levels) around 5–10 dB^1^,^2^,^3^,^4^. To move beyond this limitation, spectrometer designs which admit post-fabrication calibration and correction in the digital domain, e.g. Fourier and speckle pattern based spectrometers, have recently gained interest^5^,^6^,^7^. Speckle-based spectrometers are particularly attractive because it is relatively easy to achieve very high photon collection efficiency and correspondingly high sensitivity^8^,^9^,^10^. In these spectrometers, a scattering element is used to spatially disperse the input into a random pattern, which is then compared to previously measured calibration data for spectral reconstruction.

Multimode waveguides are appealing scattering elements because they have very low loss. As with other types of integrated spectrometer, there remains a design trade-off between footprint and bandwidth. Though in principle a multimode spectrometer could be used over the whole range in which the waveguide loss is low, the spectrum can only be faithfully reconstructed over a sub-interval of that range. The size of this interval is roughly equal to the product of the number of statistically independent spatial speckles and the spectrometer resolution. For a fully integrated spectrometer, where the speckle pattern is detected by a photodiode array, the number of photodiodes and associated fan-out may ultimately limit the footprint. Here and in ref. 11, we demonstrate a parallelization scheme which uses on-chip mode launchers to vary the input launch condition. In this configuration, the bandwidth scales with the number of spatial channels squared rather than linearly. As proof of concept, we show a spectrometer with 2 GHz resolution and 250 GHz range occupying an area of only 3.5 mm^2^; without parallelization, the usable range of this spectrometer would be closer to 22 GHz. We also show that the transmission matrix of this spectrometer possesses a self-regularising property that enables doubling of the bandwidth to 500 GHz in the presence of a sparse input signal, without any *a priori* knowledge of the signal properties. This enables robust spectral reconstruction across a variety of operating conditions, in contrast to approaches based on explicit assumptions of sparsity^12^,^13^.

## Results

### Device design and characterisation

**Figure 1:**
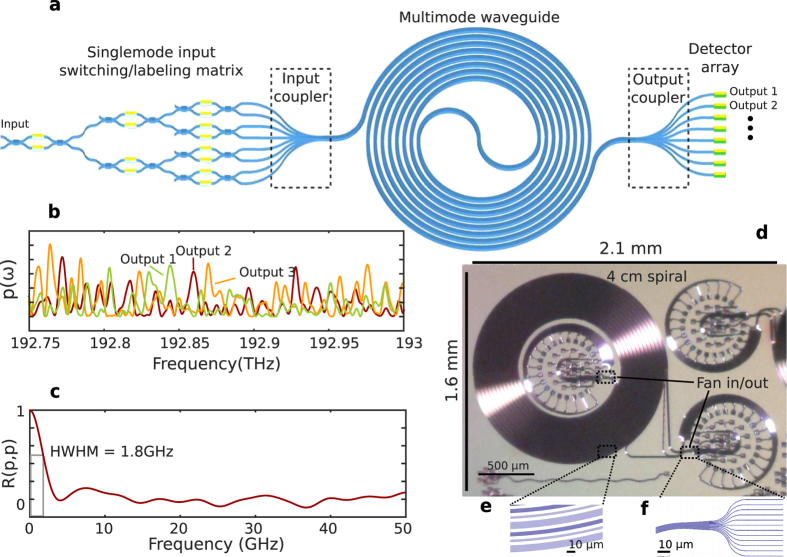
Spectrometer design and response. (**a**) Schematic including input switch matrix and integrated photodiodes. (**b**) Measured speckle pattern **p**(*x*;*ω*) at three different positions. (**c**) Mean autocovariance of **p**(*ω*). (**d**) Micrograph of fabricated chip. (**e**) Mask view of the bus waveguide. Waveguides that are not part of the device under test are lighter in colour. (**f**) Mask view of the fan in/out. The multimode input/output enters at left, tapers out, and is butt-coupled to 12 singlemode waveguides.

The device is based on a multimode waveguide with fixed singlemode input and output couplers, as illustrated in Fig. 1(a). The main purpose of the multimode waveguide is to produce measurably different output speckle patterns for different input wavelengths. The speckle pattern at the output depends on the relative phases of the excited modes, and thus changes with exp(*jω*Δ*τ*_*g*_), where Δ*τ*_*g*_ is group delay difference between the fastest and slowest principal modes of the waveguide^14^. Spectral reconstruction is performed by comparing a measured speckle pattern to a set of previously measured calibration data. Because the speckle pattern at the output is also a function of the launch condition, or the relative phases and amplitudes of the set of excited modes, it is important to ensure consistency between the launch used to calibrate the device and the launch used to measure an unknown spectrum. The input coupler is integrated on-chip to provide this long-term stability. The speckle imaging system, or output coupler, is also integrated for mechanical robustness.

Figure 1(d) shows the fabricated chip. The silicon microstrip multimode waveguide was 4.85 mm × 220 nm and 4 cm long. It supports 12 transverse electric modes and has a mode-averaged propagation loss around 0.9 dB/cm. The chip contains three non-interacting nested multimode waveguides that all have different widths as illustrated in Fig. 1(e); the only effect of the other two waveguides is to increase the total chip footprint. All three follow the path of an Archimedean spiral with 4 mm spacing between adjacent waveguides. Both the input and the output couplers were formed by adiabatically tapering the waveguide to 7.1 mm and then butt-coupling 12 singlemode waveguides to the widened output as shown in Fig. 1(f). The speckle pattern measured this way is can thus be considered a 12 × 1 pixel image. Though this is small compared to a conventional digital camera, it is sufficient to fully capture the range of possible behaviours, as there should only be 12 statistically independent output speckles^14^. The chip was fabricated by imec in a multiproject wafer run, and the singlemode waveguide dimensions and minimum spacing were given by the associated design rules. The singlemode waveguides were routed to TE-only grating couplers for characterisation. The total insertion loss of the input/output coupling scheme was 11 dB at peak transmission and is dominated by the ~6 dB loss of the grating couplers. These grating couplers could be replaced with photodiodes at the output and a switching matrix at the input for a complete on-chip system with lower loss, as illustrated in Fig. 1(a).

The achievable resolution *δω* of the reconstructed spectrum depends on how much the output pattern changes as a function of input wavelength. It has been shown^10^ that the resolution, *δω*, is approximately equal to the half width at half maximum of the autocovariance function of **p**(*ω*), where **p** is a vector of optical intensity values representing the speckle pattern and the normalized autocovariance is defined as:


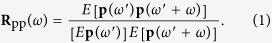


Figure 1(b) shows the measured speckle intensity for three outputs (pixels) as a function of optical frequency, and Fig. 1(c) shows the mean autocovariance function of the pattern.

Given an output speckle pattern **p** and calibration matrix **T**, the input spectrum ***s*** can be estimated using


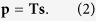


If only a single launch is considered, **p** will be an *N* × 1 vector and **T** will be *N* × *B*, where *N* is the number of outputs and *B* is the number of frequency bins considered. If instead *M* distinct launch conditions are considered, a **p** will be *MN* × 1 and **T** will be *MN* × *B*. The bandwidth over which ***s*** can be accurately reconstructed depends on the properties of the calibration matrix **T**. In particular, the bandwidth is approximately equal to the product of the resolution and the number of nonzero singular values^10^ of **T**, which can be interpreted as the number of statistically independent spatial channels. For a multimode spectrometer with a single unique launch condition, the number of distinct speckles is equal to the number of modes supported by the waveguide, *N*^14^. One degree of freedom is lost due to power conservation, so that at most *N* − 1 spatial channels are statistically independent. This gives rise to an unfavourable scaling rule for device footprint because these spatial channels have a minimum size. However, changing the input launch condition can produce a second, statistically independent, pattern, without any inherent increase in the footprint. For most practical launch cases, this can be repeated up to *N* times, so that the bandwidth can be extended to (*N* − 1)^2^*δω*.

**Figure 2:**
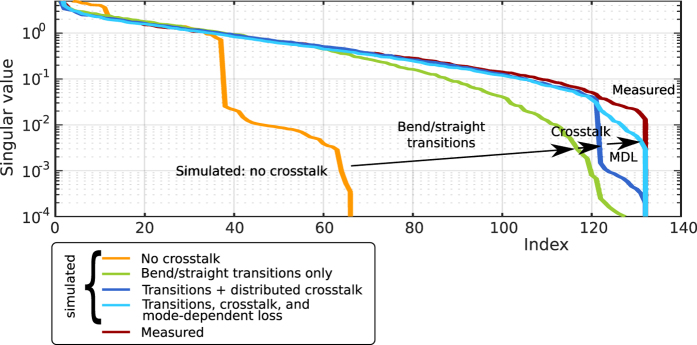
Simulated and measured singular values of T for the multimode waveguide spectrometer. Matrices were normalised before calculating the singular value decomposition such that only their relative values are meaningful. Singular values are ordered by magnitude; the singular value with index 10 is the 10th largest singular value.

In order for the calibration and measurement conditions to match consistently, all launches used must be repeatable, which in effect means that an on-chip singlemode-to-multimode transition is required. The butt-coupling method discussed above fulfils this criteria, but using the same design for both the input and the output reduces the number of statistically independent input launch conditions to *N*/2 due to symmetry. Simulated and measured singular values of ***T*** for this waveguide geometry with 12 input launch conditions and 12 outputs are shown in Fig. 2. The speckle calibration matrix was normalised to have a Frobenius norm of 1 before computing the singular value decomposition in order to facilitate comparison between different cases. The singular values are shown in descending order. In the case of symmetric singlemode-to-multimode transitions, **T** has only 66 nonzero singular values. Including intermediate mode coupling at the bend-straight transitions near the input and output increases the number of nonzero singular values to 121 by breaking this symmetry. However, these decay at a rate much faster than that observed in the experiment. Including mode-coupling at an intermediate point in the 4 cm long waveguide brings the simulation results closer to the experimental ones. The simulated curve assumes a single coupling event corresponding to crosstalk of 20 dB (each mode loses on average 1% of its power to the other modes). Mode-dependent loss (MDL) produces an additional 12 nonzero singular values.

### Spectral reconstruction

The device was calibrated using a swept tunable laser source. The wavelength response of each input-output pair was measured sequentially, with each measurement forming a row of the speckle calibration matrix **T**. Figure 1(b) shows three such rows. Different pairs were accessed by mechanically translating a single input and a single output fiber, but this process could easily be replicated using switches and photodiodes, as illustrated in Fig. 1(a). The columns of **T** were re-scaled so that the total output power for each input location and frequency would sum to 1; this had the effect of forcing the numerically zero singular values of **T** to true zeros, and also removes information about the frequency dependence of the grating coupler response. The primary source of error in the calibration procedure was the absolute wavelength repeatability of the laser. Errors in the calibration matrix decrease the resolution and power accuracy of the spectrometer.

Even when the bandwidths of **T** and ***s*** are restricted to the useful range of the spectrometer, the calibration matrix will be ill-conditioned. Statistical dependence arises between rows due to power conservation, and between adjacent columns due to the slow decay of the autocorrelation of the frequency response (or intentional over-sampling of the frequency response). The result of this ill-conditioning is that when equation (2) is solved using the Moore-Penrose pseudoinverse, the reconstructed spectrum contains non-physical high-frequency oscillations. This problem is typically solved using some form of regularisation, most commonly truncated singular value decomposition^8^,^10^,^15^ or Tikhonov regularisation^9^. These two techniques are closely related and perform similarly. Tikhonov regularisation consists of minimising the penalised least-squares cost function:


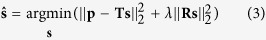


where *λ* and **R** are a scalar tuning parameter and design matrix, respectively. Several suitable choices of **R** are available; for dense spectra, the identity matrix, smoothing matrices, and a weighted (low-pass) Fourier operator all produce similar values for 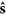. A positivity constraint on the reconstructed spectrum 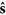 has also been shown to improve performance by ruling out non-physical spectra^10^,^15^. If the measured spectrum is known to be sparse, 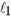 regularisation (compressive sensing) can also be used^12^,^13^,^16^. In this case, the 2-norm in the second term in equation (3) is replaced with the 1-norm and the solution is found using the lasso rather than by direct construction. It should be noted that equation (3) assumes that the spectrum is best described in the delta function basis. However, this is not strictly necessary; if 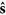 is known to be sparse in some other basis (e.g. the Haar basis), this can be taken into account by inserting an appropriate linear transformation into the equation^17^.

Regularisation methods typically require selection of a hyperparameter; either *λ* or the minimum singular value. This is roughly analogous to choosing the resolution bandwidth in a traditional grating-based spectrometer. As in that case, the optimum value for the hyperparameter depends on the measured spectrum in addition to the spectrometer properties. A number of strategies exist for hyperparameter selection^18^. These all increase total computation time because they multiply the total number of calculations involved to obtain a single spectrum by the number of hyperparameter values considered. The most effective method proved to be minimising the error using a sequence of synthesised (and therefore known) test spectra and associated speckle patterns. By using both dense and sparse training spectra, generally applicable hyperparameters could be chosen. This has the additional benefit of not requiring extra computation if the spectrometer is used to measure unknown spectra.

#### Dense spectra

**Figure 3:**
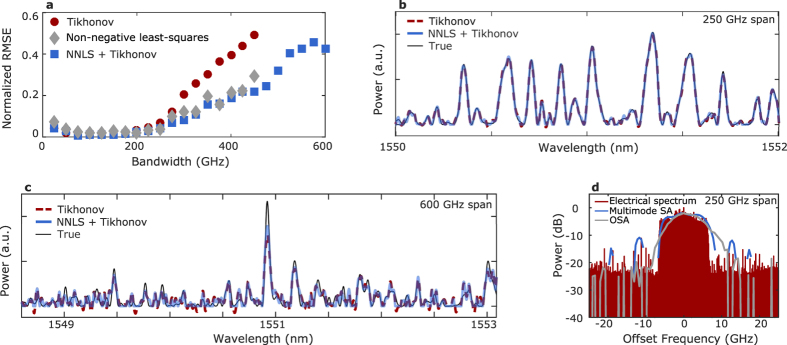
Spectrometer performance for dense spectra. (**a**) Root mean squared error (normalised to mean power level) as a function of calibration bandwidth for three different regularisation methods. Each point was averaged over ten test inputs. Reconstructed test input spectra over a (**b**) 250 GHz bandwidth and (**c**) 600 GHz bandwidth. (**d**) Reconstructed spectrum of a 12.5 GBd Nyquist-shaped QPSK signal. The line is not visible in regions where the estimated power is zero. Though only the central 50 GHz are shown, the spectrum was calculated over a 250 GHz bandwidth.

To quantitatively evaluate the performance of the spectrometer for dense input spectra, we synthesised random test spectra using a procedure described in the Methods section and calculated the mean-squared error between the known input and the reconstruction from **Ts**. No noise was added to **Ts** to emulate additional noise at the detector, but **T** was measured experimentally and so the results are affected to some extent by noise. The root mean squared error (RMSE) between the test spectrum and the spectrum reconstructed using Tikhonov regularisation, a positivity constraint only, and a combination of the two is shown in Fig. 3(a) as a function of the bandwidth of the reconstruction range. All three methods perform well from zero to 250 GHz, at which point performance starts to degrade. The non-negative least-squares (NNLS) solution only performs well over the full bandwidth when the frequency bin spacing is exactly 2 GHz. In contrast, changing the frequency bin spacing only changes the optimum hyperparameter for the Tikhonov cases. The strong performance of the NNLS solution despite the absence of an explicit penalty for oscillation indicates that the calibration matrix possesses “self-regularising” properties, most likely due to its close similarity to an equi-correlated design^19^. The NNLS+Tikhonov solution is far less sensitive to hyperparameter selection than the Tikhonov solution, which may be a consequence of this property.

An example of a test spectrum with 250 GHz bandwidth and two solutions is shown in Fig. 3(b). Though some discrepancy is calculated for this reconstruction, it is not clearly visible. Reconstructed spectra over a bandwidth of 600 GHz are shown in Fig. 3(c). Though the RMSE in Fig. 3(c) is large (1.5 dB for the NNLS + Tikhonov solution), the quality may be sufficient for some applications. The results shown in Fig. 3(a)–(c) represent the best-case scenario: no measurement noise and no error in the calibration matrix. To verify that the spectrometer can be used when realistic levels of measurement noise and calibration errors are present, the spectrum of a dual-polarisation 12.5 GBd QPSK signal with Nyquist pulse shaping was measured and is shown in Fig. 3(d) along with the spectra measured by a coherent receiver and by an optical spectrum analyser (OSA). The signal was generated using an arbitrary waveform generator at 62.5 GS/s, an external cavity laser, an I-Q modulator, and a pol-mux emulation stage; the setup was similar to the one shown in ref. 20. Because the grating couplers only couple the TE part of the input signal into the waveguide, using the spectrometer to evaluate a polarisation-multiplexed signal incurs a 3 dB penalty in insertion loss. However, this has no effect on the measured spectrum because it is the same in both polarisations. The RMSE cannot be calculated for this case, but the result is qualitatively consistent with the results in Fig. 3(a)–(c). The superior resolution of the multimode spectrometer is visible in the sharp roll-off at the edge of the signal spectrum relative to the spectrum measured using the OSA.

#### Sparse spectra

When the input spectrum is sparse in some basis, this information can be used to improve the performance of the spectrometer^12^,^13^. In particular, sparsity permits the use of 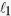 regularisation^21^, which enhances both bandwidth and resolution. To quantify the bandwidth enhancement, the dynamic range of the spectrometer was estimated using test spectra. The test inputs consisted of a series of evenly spaced tones with decreasing amplitudes. The dynamic range was defined as the ratio between the highest power tone and the tone with the least power that was recovered within 3 dB accuracy. The dynamic range, defined in this way, is approximately analogous to the crosstalk in an arrayed waveguide grating, because it represents the level at which fluctuation power is approximately equal to the signal power.

**Figure 4:**
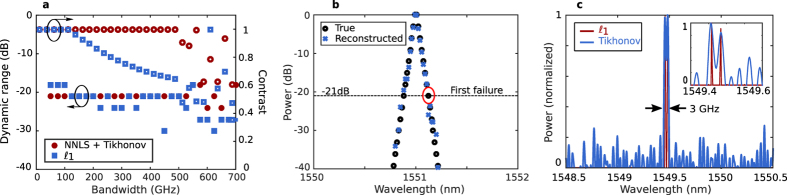
Spectrometer performance for sparse spectra. (**a**) Dynamic range and contrast for tones with 2 GHz spacing using synthesised inputs. (**b**) Example dynamic range calculation (measured calibration matrix and synthesized inputs). (**c**) Fully experimental recovered spectrum of two external cavity lasers spaced by 3 GHz. Inset: zoom in.

Figure 4(a) shows the dynamic range and contrast as a function of calibration matrix bandwidth for tone spacings of 2 GHz. Figure 4(b) shows an example of a test signal recovered with 20 dB dynamic range. Points between tones typically go to zero and thus are not visible in the figure. The contrast is defined as 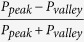. Both 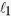 regularisation and NNLS+Tikhonov provide a dynamic range around 20 dB over a 500 GHz bandwidth. However, a resolution penalty appears in the 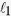 case for bandwidths greater than 150 GHz. As again the information shown in Fig. 4(a) and (b) represent the best-case behavior, we also measured performance experimentally. The reconstructed spectrum of an input consisting of two lasers with 3 GHz frequency spacing is shown in Fig. 4(c). For the spectrum reconstructed using only Tikhonov regularisation, the trade-off between resolution and oscillations is clear, whereas 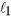 regularisation is able to produce an oscillation-free spectrum with clearly separated lines. The spectrum reconstructed using both NNLS and Tikhonov regularisation is indistinguishable from the 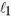 solution and not shown. The contrast is 1 for both methods up to the 700 GHz calibration bandwidth. The performance in this case is better than the performance shown in Fig. 4(a) because the input is more sparse and the tone spacing is larger.

## Discussion

We have demonstrated a multimode waveguide-based spectrometer scheme that has a favorable trade-off between performance and footprint. The bandwidth has been shown to scale with (*N* − 1)^2^ rather than linearly with the number of modes *N*. Though the photon collection efficiency of the fabricated device is high, speckle-based spectrometers suffer from an inherent sensitivity penalty^22^ relative to grating-based ones in the low-photon regime due to the spreading of the input signal over several detectors. For a given bandwidth, the parallelised scheme proposed here would mitigate this penalty by spreading the signal over fewer detectors. The cost of parallelising is measurement time, but the device was fabricated on an integration platform where broadband switches and photodiodes with bandwidths in excess of 25 GHz are available, so this could most likely be minimised.

We have also shown that the combination of a positivity constraint and Tikhonov regularisation can provide excellent performance in reconstructing an input spectrum from the measured speckle pattern for both dense and sparse spectra. This provides an appealing option for spectrum reconstruction in speckle-based devices generally, as the same method can be used even when no *a priori* knowledge of the input is available. It remains to be seen whether the best achievable dynamic range and lowest RMSE are related to properties of the waveguide itself (as is the case with the crosstalk in an arrayed waveguide grating), or if they arise from avoidable or correctable errors in the calibration process^23^. The best performance that can be obtained using the simulated calibration matrix is better than what has been demonstrated here, but this could be due to an effect not included in the device model. If the error does truly originate in the calibration process, this suggests an algorithm for correcting the calibration matrix without resorting to an external stable reference.

## Methods

### Optical simulation

The total response of the device was simulated using transmission matrices. The modes of the straight waveguide were calculated using the method and software package described in ref. 24. The modes of the bent waveguide, and the intermediate coupling matrix, were calculated using the method in ref. 25. The matrix used to simulate distributed coupling was formed using an appropriately weighted sum of a random unitary matrix generated using the algorithm in ref. 26 and the identity matrix.

### Training and test spectra

To generate dense training and test spectra, auxiliary functions **r** were randomly generated from a Gaussian process^27^ with a squared exponential kernel and a tunable characteristic length. The test spectrum ***s*** was generated by squaring the auxiliary function in order to enforce a positivity constraint. This process is summarised in psuedo-code below. To calculate the RMSE, the characteristic length was set to 4 GHz, resulting in spectra that varied quickly enough to penalize regularization methods with overly aggressive smoothing.

### Numerical methods for regularisation

The non-negative least-squares solution was calculated using the fast algorithm in ref. 28. The use of this method was inspired by ref. 10, but the algorithm suggested in that work cannot produce spectrum values that are exactly zero, so the effect of applying a positivity constraint in this way is most likely different. Tikhonov regularization was added by completing the square:


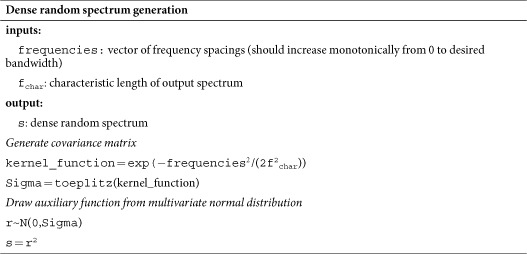



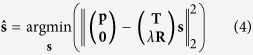


and solving the augmented problem using the same algorithm. The 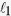 solution was calculated using Matlab’s bulit-in lasso function. Setting the convergence threshold appropriately was found to be very important in achieving good performance.

## Additional Information

**How to cite this article**: Piels, M. and Zibar, D. Compact silicon multimode waveguide spectrometer with enhanced bandwidth. *Sci. Rep.* **7**, 43454; doi: 10.1038/srep43454 (2017).

**Publisher's note:** Springer Nature remains neutral with regard to jurisdictional claims in published maps and institutional affiliations.
